# Wastewater use in algae production for generation of renewable resources: a review and preliminary results

**DOI:** 10.1186/2046-9063-9-2

**Published:** 2013-01-05

**Authors:** Omatoyo K Dalrymple, Trina Halfhide, Innocent Udom, Benjamin Gilles, John Wolan, Qiong Zhang, Sarina Ergas

**Affiliations:** 1 Civil & Environmental Engineering Department, University of South Florida, Tampa, FL, 33620, USA; 2 Chemical & Biomedical Engineering Department, University of South Florida, Tampa, FL, 33620, USA

## Abstract

Microalgae feedstock production can be integrated with wastewater and industrial sources of carbon dioxide. This study reviews the literature on algae grown on wastewater and includes a preliminary analysis of algal production based on anaerobic digestion sludge centrate from the Howard F. Curren Advanced Wastewater Treatment Plant (HFC AWTP) in Tampa, Florida and secondary effluent from the City of Lakeland wastewater treatment facilities in Lakeland, Florida. It was demonstrated that a mixed culture of wild algae species could successfully be grown on wastewater nutrients and potentially scaled to commercial production. Algae have demonstrated the ability to naturally colonize low-nutrient effluent water in a wetland treatment system utilized by the City of Lakeland. The results from these experiments show that the algae grown in high strength wastewater from the HFC AWTP are light-limited when cultivated indoor since more than 50% of the outdoor illumination is attenuated in the greenhouse.

An analysis was performed to determine the mass of algae that can be supported by the wastewater nutrients (mainly nitrogen and phosphorous) available from the two Florida cities. The study was guided by the growth and productivity data obtained for algal growth in the photobioreactors in operation at the University of South Florida. In the analysis, nutrients and light are assumed to be limited, while CO_2_ is abundantly available. There is some limitation on land, especially since the HFC AWTP is located at the Port of Tampa. The temperature range in Tampa is assumed to be suitable for algal growth year round. Assuming that the numerous technical challenges to achieving commercial-scale algal production can be met, the results presented suggest that an excess of 71 metric tons per hectare per year of algal biomass can be produced. Two energy production options were considered; liquid biofuels from feedstock with high lipid content, and biogas generation from anaerobic digestion of algae biomass. The total potential oil volume was determined to be approximately 337,500 gallons per year, which may result in the annual production of 270,000 gallons of biodiesel when 80% conversion efficiency is assumed. This production level would be able to sustain approximately 450 cars per year on average. Potential biogas production was estimated to be above 415,000 kg/yr, the equivalent of powering close to 500 homes for a year.

## Introduction

The United States (US) imports about 57% of the petroleum it consumes. Among all sectors, transportation accounts for 72% of all petroleum consumption [1]. As energy consumption increases, the US dependence on foreign oil will also increase and compete heavily with the energy demands of rapidly growing economies such as China, India and Brazil. This will place tremendous pressure on global oil production and may decrease energy security. In addition, the wide and sustained use of petroleum-based fuels has been implicated as a major cause of increased atmospheric greenhouse gases, which may contribute to global climate change [2]. These challenges have sparked the quest for alternative energy sources to serve as viable replacements to reduce dependence on fossil fuels and improve environmental sustainability. Among the many options, microalgae are receiving enormous attention as a source for the production of biofuels. Model estimates from Pacific Northwest National Laboratory have suggested that algal biofuels (particularly biodiesel) have the potential to meet as much as 17% of the transportation fuel demand [3]. Microalgae oil production per unit area of land far exceeds other oil crops such as corn, soybean, coconut, and oil palm by as much as 2–3 orders of magnitude [4]. Furthermore, they do not compete for arable land and can be produced year-round in suitable climates. They also grow much faster than traditional crops (doubling time can be as fast as 24 hours) and are likely to recover more quickly from adverse effects [5,6].

Large-scale commercial production of algae, however, is potentially more costly than traditional crop production. Algae cultivation requires significant quantities of energy and water and the use of off-site generated carbon dioxide. One energy intensive process, for example, is the harvesting of the algal biomass, which can account for as much as 30% of the total cost of production [7-11]. In addition, water and nutrients are among the most critical variables in algal production [10,12]. Fortunately, algae can be grown in both fresh water and seawater depending on species, but nutrient costs can be substantial. The main nutritional requirements for algal growth are nitrogen, phosphorous, and a number of micronutrients including potassium [5]. Algae take up these nutrients along with CO_2_ and produce biomass via photosynthesis. Various combinations of fertilizers maybe used, including common field crop N-P-K fertilizer, but the associated costs can sometimes exceed the value of the final algae products [10].

For algal biofuels to achieve their full potential, inputs to algal cultivation must be inexpensive allowing for the economical mass production of feedstock. A convenient and cheap source of nutrients is municipal, industrial and agricultural wastewaters. Nutrient removal is an important aspect of wastewater treatment because rich nutrient streams discharged into natural water bodies can result in eutrophication. Furthermore, centrate (a nutrient-rich effluent stream from the anaerobic digestion process) is generally recycled to the head of the wastewater treatment plant and can increase the cost and destabilize the overall treatment process due to phosphorus accumulation. Since algae are known to grow in wastewater, a possible synergistic solution is to co-locate and integrate algal production with treatment of nutrient-rich wastewater and utilization of CO_2_ from power plant flue gas. This approach essentially reduces the cost of algal production, while preventing eutrophication and mitigating CO_2_ emissions [13-16].

Florida, and particularly the Tampa Bay area, has been identified as an ideal location for the development of algal feedstock and biofuel production because it receives significant sunshine, and demonstrates a relatively uniform seasonal evaporation loss compared to many other areas of the country [3]. The latter is particularly important for open pond cultivation systems that lose significant amounts of water via evaporation. In this study, wastewater use for algae production is reviewed, particularly for renewable energy generation. A preliminary assessment of the potential to produce algal feedstock from wastewater is presented for two Tampa Bay cities. These include the City of Tampa and the City of Lakeland. All the wastewater from the City of Tampa is treated at the Howard F. Curren Advanced Wastewater Treatment Plant (HFC AWTP). HFC AWTP has a designed average daily flow capacity of 96 million gallons per day (MGD) and employs high-purity oxygen aeration for biochemical oxygen demand (BOD) removal followed by nitrification and denitrification. Lakeland’s municipal wastewater is treated by two traditional wastewater treatment plants and the secondary effluent is released into a 1,400-acre wetland treatment system (WTS) to achieve permissible nutrient reduction levels. The average daily flow rate into the wetland is 5.2 MGD. The WTS consist of a series of wetland cells connected by engineered discharged structures. Effluent from the WTS is discharged to the Alafia River. A wide cross-section of freshwater algal species thrives in the WTS.

Most of the electricity supplied to the Bay Area comes from Tampa Electric Company (TEC), which has a power plant located about 15 miles south of the Lakeland WTS and another plant across from the HFC AWTP. Together, these two power plants emit approximately 5.5 million metric tons of CO_2_ annually. Further, to lessen the burden on scarce freshwater resources, TEC and the City of Lakeland entered into a reclaimed water agreement in 2009 that allows TEC to use reclaimed effluent from the WTS commencing at the end of 2012. TEC will install a water treatment system to ensure that the effluent meets its cooling water standards.

The location of these facilities presents a potentially viable opportunity to explore synergy for algal feedstock production using wastewater and industrial CO_2_. A preliminary assessment was made to determine the quantity of algal feedstock that can be generated. The analysis was guided by experimental work on the growth of algae in enclosed bench-scale photobioreactors. The aim was to assess algae growth rate, nutrient uptake and lipid production using anaerobic digestion centrate from HFC AWTP and the Lakeland WTS.

### Experimental methods

#### Inoculum collection and scale-up

Wild-type algae were harvested from a secondary clarifier at the HFC AWTP in Tampa, Florida. Samples were transferred to 1-L flasks and bubbled with 2% CO_2_ in air during an 18-hr light/dark cycle under artificial light conditions of 310 μmol m^-2^ sec^-1^. Anaerobic digestion sludge centrate from the same facility was used as the scale-up medium after removal of suspended matter with a filter cloth. There were no nutrient additions to the centrate. Inoculum was grown until the culture biomass was 2 g dry wt L^-1^ as determined by total suspended solid (TSS) analysis with 5 mL algae suspension according to the standard method [13]. University of Florida Environmental Biotechnology Laboratory analyzed samples and determined that the main algal species were *Chlorella* sp. and *Scenedesmus* sp.

#### Photobioreactor setup and operation

**Figure 1:**
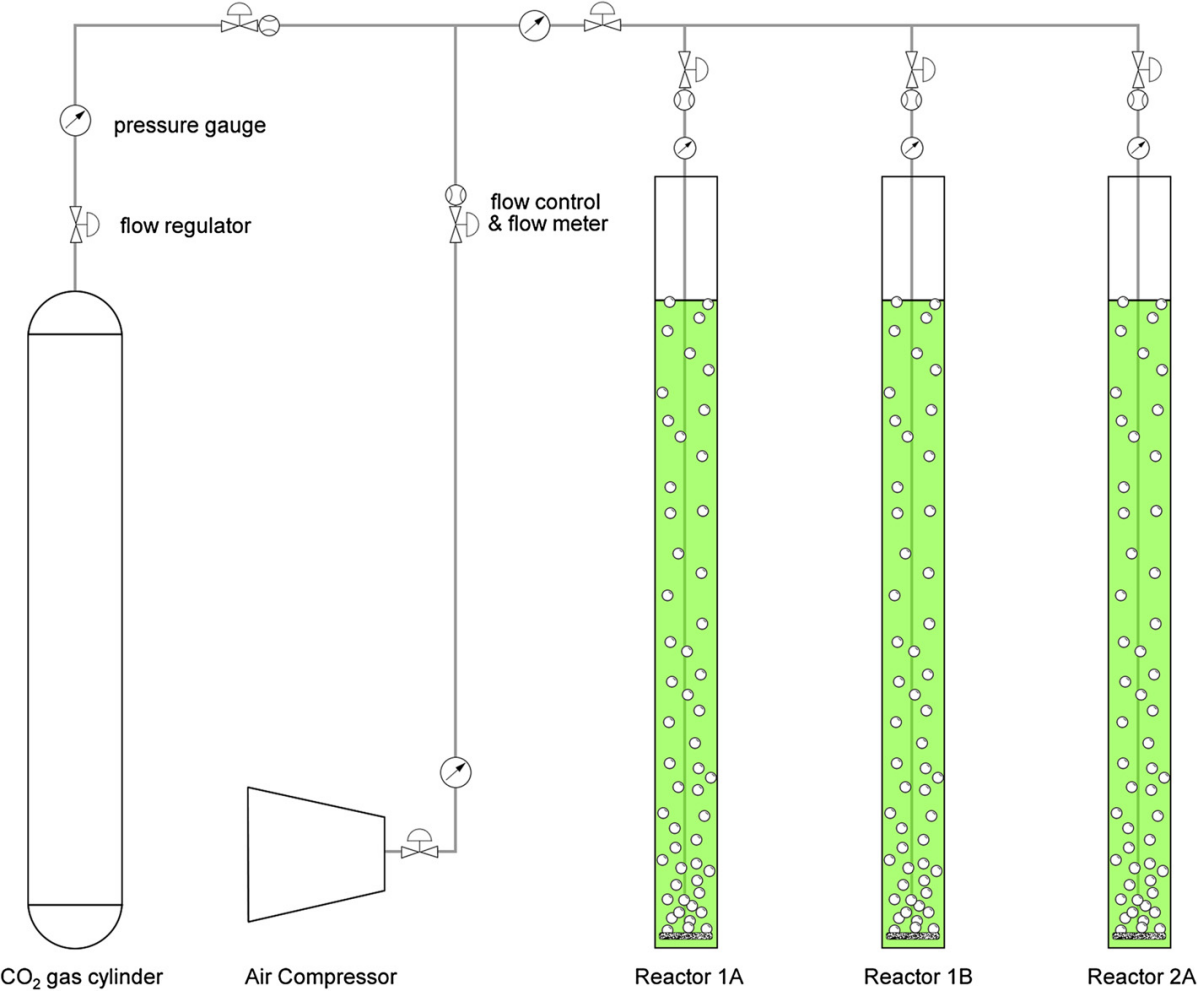
Schematic of bench-scale tubular plastic photobioreactors located in greenhouse at the University of South Florida, Tampa, FL and operated under natural light conditions.

The algae cultivation setup consisted of three tubular polyethylene photobioreactors (obtained from the Norwegian University of Life Sciences, Norway), which were housed in a greenhouse at the Botanical Gardens of the University of South Florida in Tampa, Florida. Figure 1 shows the setup of the photobioreactors, which began operation in February 2011. The reactors were 237.13 cm high with a diameter of 12.32 cm. They were each operated at a volume of 7 liters. Air containing 2% CO_2_ was bubbled through the reactor using coarse bubble diffusers to provide inorganic carbon for photoautotrophic growth, as well as mixing. The gas flow rate was maintained at 0.5 L min^-1^. The reactors were operated on a semi-continuous basis with a mean cell retention time of 7 days.

Each day, 1 L of the reactor volume was replaced with centrate collected from the HFC AWTP. The nutrient content of the centrate was analyzed prior to feeding the reactors. A data-logger (Onset® HOBO U12) was used to record irradiance, ambient temperature, culture temperature and relative humidity every 15 minutes.

A 1-L batch reactor was also operated with wetland water from the City of Lakeland WTS. The WTS contained a native population of algae, whose diversity was previously analyzed by GreenWater CyanoLab (Palatka, FL) and shown to include *Bacillariophyta, Chlorophyta* and *Cyanobacteria* groups. Air with 2% CO_2_ was fed to the reactor in like manner as the plastic reactors. A low-nutrient media was maintained by semi-continuous addition of 50 mL of 22.5 mg L^-1^ K_2_HPO_4_ and 60.71 NaNO_3_ to the batch reactor. The batch was operated for 3 weeks. Similar nutrient analyses were performed as previously described. All nutrients used in the study were obtained from Sigma Aldrich (St. Louis, MO).

#### Pretreatment and wastewater characterization

Anaerobic digestion sludge centrate was collected once weekly and filtered with a fabric to remove coarse bio-solids. Total nitrogen (TN), ammonia and total phosphorous (TP) content were determined. To avoid death of the culture when the centrate nutrient content was very low, the TN concentration in the feed was maintained between 200–250 mg L^-1^ by addition of (NH4)_2_SO_4_. Table 1 provides details of the nutrient content of the centrate.

**Table 1:** Nutrient content of centrate used as growth media for mixed algae species

**Parameter**	**Range**
Total nitrogen	200 – 250 mg L^-1^
Total phosphorous	2 – 75 mg L^-1^
Ammonia	100 – 250 mg L^-1^

#### Biomass and nutrient monitoring

Measurements of TSS and pH were performed daily. Nutrient removal analyses were performed every week for TN, ammonia (NH_3_), nitrate (*NO*_3_^−^), TP and chemical oxygen demand (COD) according to Standard Methods [17]. TSS was determined by filtering a 5-mL algae suspension followed by drying in an oven for 24 hours.

#### Lipid content analysis

The algal lipid content was determined according to the method by Bligh and Dryer [18]. A sample of algae suspension was centrifuged at 3,800 rpm for 10 minutes to obtain a concentrated algae paste. The dry weight (*w*_*d*_) of the paste was determined gravimetrically after drying at 60°C. A 2-mL sample of algae solution was mixed with 4 mL of a 2:1 methanol/chloroform solution in a glass vessel. The suspension was left for 24 hours. Thereafter, 1 mL of chloroform was added and the solution was mixed on a vortex for 1 min. 2 mL of water was then added and the mixture was again agitated for 2 min. The layers were separated by centrifugation at 2,000 rpm for 10 min. The lower layer was extracted with a glass syringe and filtered through a Whatman no. 1 filter into a previously weighed glass vessel (*w*_1_). The solvent was dried in a water bath at 98°C and the vessel was weighed again (*w*_2_) to obtain the lipid content of the sample as;

(1) $$\mathrm{lipid}\;\mathrm{content}=\frac{W_{2}-W_{1}}{W_{d}}x100\%$$

## Results

### Light conditions

**Figure 2:**
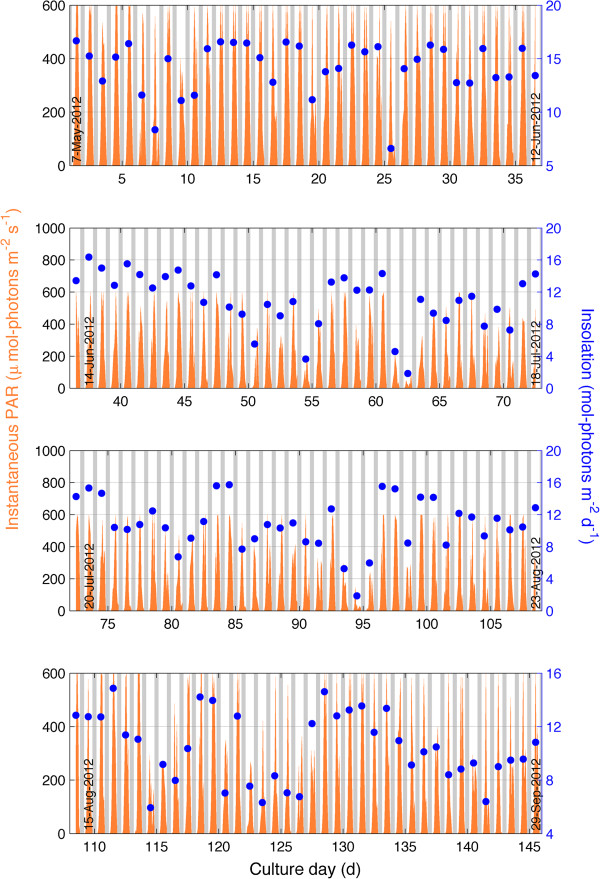
**Instantaneous PAR and daily insolation during cultivation period from May to September 2011.** Data was recorded in the greenhouse at University of South Florida, Tampa, FL. Daily insolation was obtained by integrating PAR over day.

The experiment was conducted in the summer from May 7, 2011 to September 30, 2011 in greenhouse conditions at the University of South Florida, Tampa, Florida. An evaporative cooling system kept peak daily ambient temperatures in the greenhouse below 40°C. Figure 2 shows the instantaneous PAR and daily integrated insolation for the period of cultivation. Daily insolation was highest in the early summer months (May-July) averaging 12 mol-photons m^-2^ d^-1^. During the latter period of cultivation (August-September), mean daily insolation fell to 10 mol-photons m^-2^ d^-1^. Daily peak instantaneous PAR was *ca*. 600 μmol-photons m^-2^ s^-1^.

### Temperature and pH

**Figure 3:**
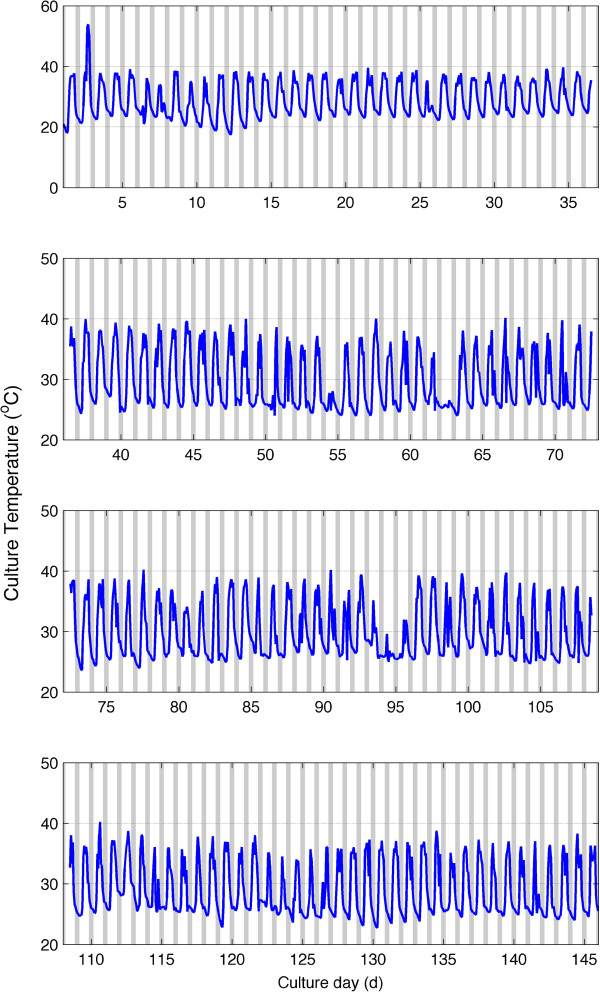
Culture temperature profile recorded from May-September 2011 during cultivation of wild algae grown on anaerobic sludge centrate in Tampa, FL.

**Figure 4:**
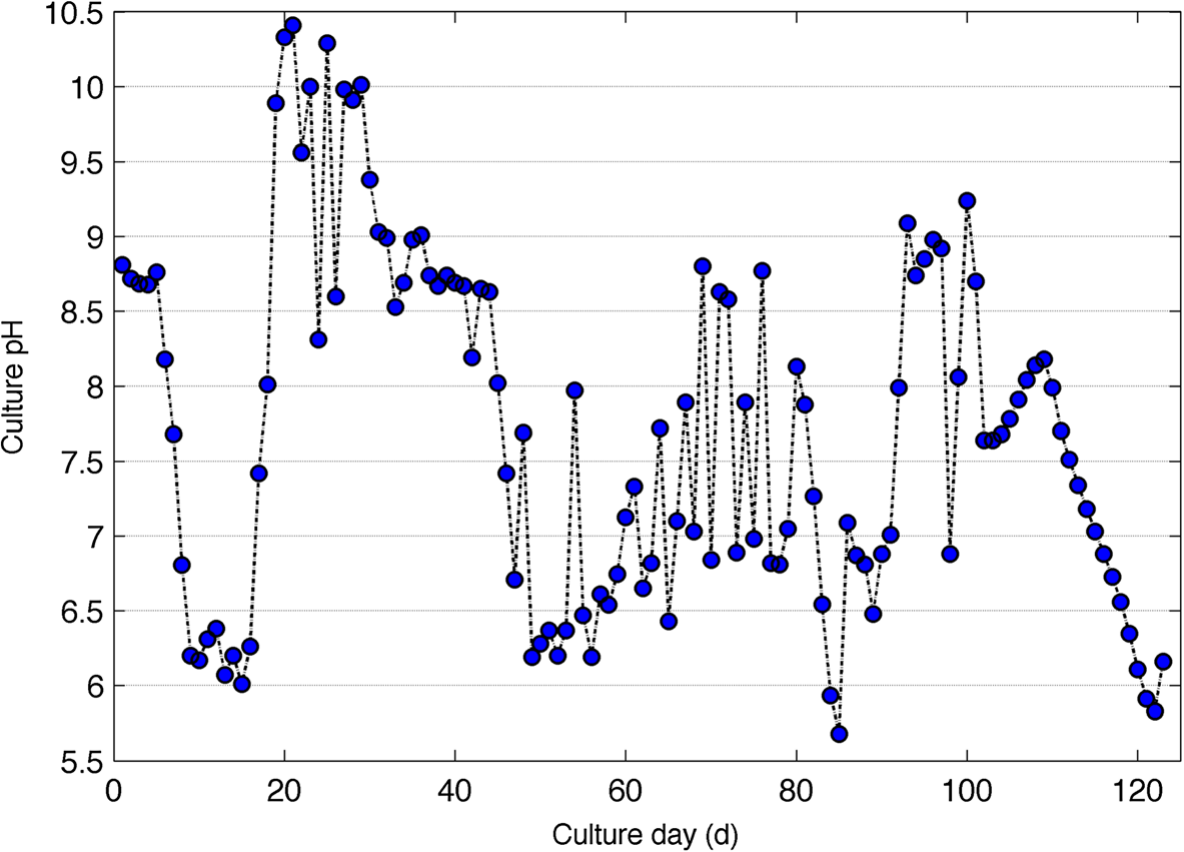
**Daily culture pH for algae grown on anaerobic sledge centrate from the Howard F.** Current Advanced Wastewater Treatment Plant, Tampa, FL. pH measurement was taken once a day and therefore do not capture any diurnal variations.

Culture temperature for the duration of the experiment is shown in Figure 3. Mean culture temperature was 29.2°C. Peak daily culture temperatures remained mostly below 40°C. Diurnal temperature changes were on average 13°C for the period of cultivation. Changes in pH are shown in Figure 4 and were more variable ranging between 6 and 9. There was an excursion of pH above 9 from days 20 to 30.

### Biomass development and production rates

**Figure 5:**
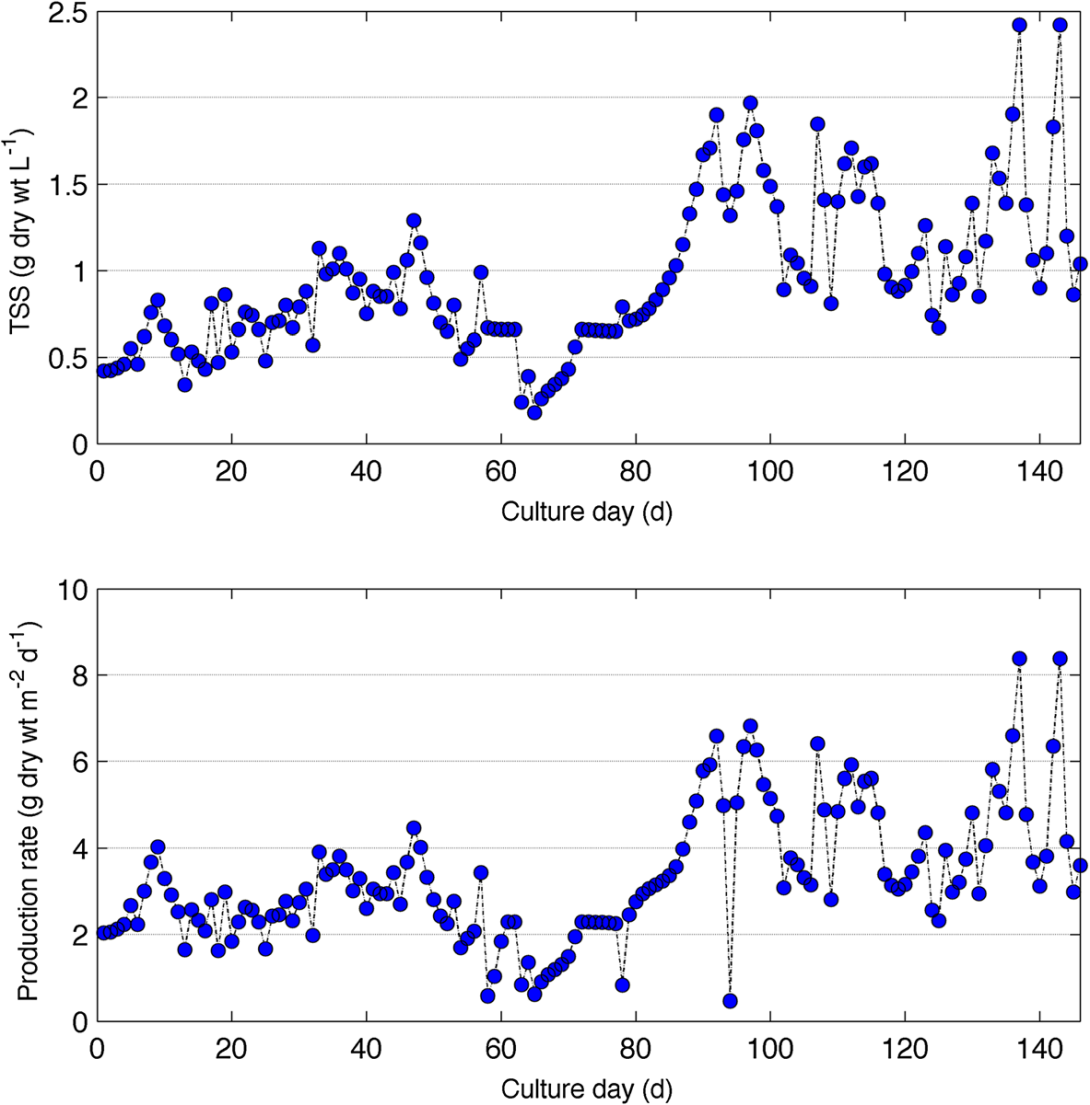
**Growth (top) and areal biomass productivity (bottom) of mixed algal species grown on anaerobic sludge centrate for the period May to September 2011 in Tampa, FL.** Productivity calculations are based on reactor illuminated surface area.

Microalgae biomass development in the photobioreactors is shown in Figure 5. Standing biomass concentration during the first 80 days of operation was 0.75 g dry wt L^-1^. Air diffusers were replaced on day 80 and resulted in improved mixing and an associated doubling in the standing biomass. Steady state dry biomass concentration remained below 2 g dry wt L^-1^. Photobioreactor areal production *P* (g dry wt m^-2^ d^-1^) was calculated based on illuminated surface area *A* (m^-2^) according to equation 2.

(2) $$P=\frac{\mathrm{QC}}{A}$$

where Q is the daily flow rate (L d^-1^) and C the algae biomass concentration (g dry wt L^-1^). The mean production rate for the first 80 days was 2.5 g dry wt m^-2^ d^-1^, which increased to 4.5 g dry wt m^-2^ d^-1^ for the last 45 days. The maximum sustained production rate was 7 g dry wt m^-2^ d^-1^ for one week. The areal productivity of the batch culture with Lakeland WTS algae was approximately 0.5 g dry wt m^-2^ d^-1^ (data not shown). For the tubular reactors, the most active growth period occurred from days 87–100, after the diffusers were replaced.

### Nutrient uptake

**Figure 6:**
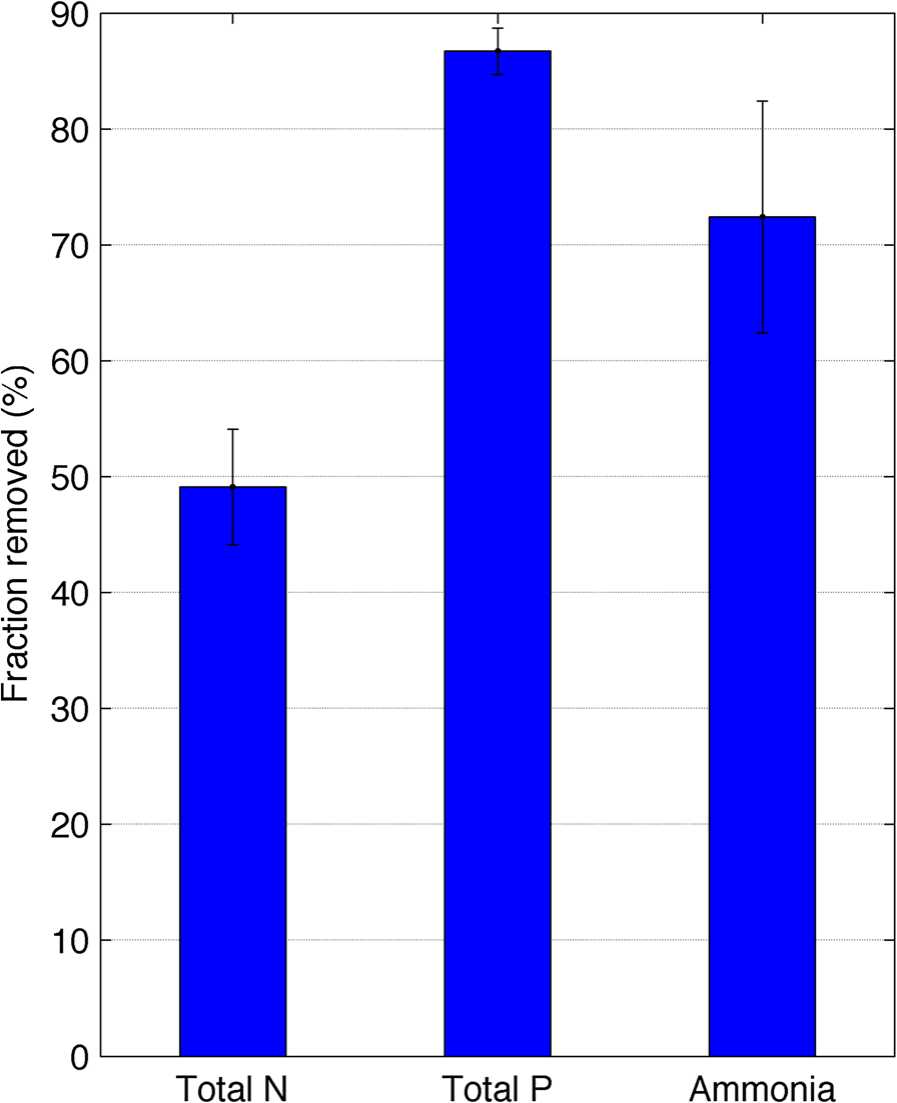
**Fraction of nutrients taken up by mixed algae species grown in enclosed photobioreactor from centrate medium.** Nutrient uptake was determined from the difference between filtered and unfiltered samples.

The fraction of nutrients taken up is illustrated in Figure 6. Nutrient uptake was determined from the difference between filtered and unfiltered samples. The latter was diluted before analysis. TN uptake was just below 60%, while 72% ammonia was taken up. Phosphorous removal was greater than 85%.

### Lipid content

Algae grown on the high strength centrate had very low lipid content (<10%) compared to the 65% lipid content of Lakeland WTS algae consortium.

## Discussion

### Algae biomass production potential from wastewater resources

This study was conducted to assess the potential of cultivating algae using wastewater as a nutrient medium. The consortium of algal species, including *Scenedesmus* sp. and *Chlorella* sp. grew favorably on anaerobic sludge centrate from the HFC AWTP. There was relatively high nutrient uptake for phosphorous and ammonia. Total nitrogen uptake was much lower because organic nitrogen was most likely not assimilated by the culture. The mean productivity obtained for the entire cultivation period was 3.3 ±1.5 g dry wt m^-2^ d^-1^. These results are similar to Woertz et al. [19] who report an algae production rate of 3 g dry wt m^-2^ d^-1^ for *Chlorella* sp. grown on wastewater. Li et al. [20] report a biomass production rate of 13 g dry wt m^-2^ d^-1^ for algae grown on centrate. Their results showed that by the end of a 14-day batch culture 94% ammonia, 89% TN and 81% TP was removed. Their system was continuously operated at 50% daily harvesting rate, compared to 14% used in this study. Zhou et al. [21] also grew algae on full strength anaerobic sludge centrate and obtained a biomass production rate of 12.8 g dry wt m^-2^ d^-1^. The lipid content reported by Li et al. [20] was ca. 11%, similar to these results. This is a downside of growing algae, especially *Chlorella* sp., in high strength nitrogen media. The caloric content which is linked to lipid production is significantly reduced [22]. In general, high lipid content is achieved when the organisms are “starved” of nitrogen [4,22,23].

### Potential application to large-scale algal production

Photobioreactor optimization can potentially increase biomass production, as observed from improving only air bubbling in this study. Improved air delivery was achieved by changing from spherical to cylindrical ceramic diffusers, resulting in better mixing. Work by Richmond [24], Richmond and Zou [25] and Qiang and Richmond [26] indicates that highly productive and efficient enclosed algal systems can be obtained by optimizing cell density and mixing rate in relation to photon flux density, particularly when nutrients are not limited. In addition, better aeration promotes increased mass transfer allowing for the removal of oxygen, which can become inhibiting at high concentrations [19].

However, there are limits to the photosynthetic conversion of sunlight energy into algal biomass in large-scale outdoor cultures. Under light-limited growth, there is an upper limit for light conversion efficiency of a large-scale culture. In practice, this usually translates to a maximum potential yield of 30–40 g dry wt m^-2^ day^-1^ under ideal outdoor sunlight conditions for short periods and considerably less for longer durations. This indicates that the non-optimized operation in this preliminary assessment was able to achieve 10% of the maximum. However, the cultures were grown under conditions of reduced light. It is possible to cultivate algae outdoor and improve light utilization through vertical reactor orientation, while keeping peak temperature down due to mutual shading of reactors [27].

Production in high rate algal ponds (HRAP) is possible and has shown commercial production rates as high as 40 g dry wt m^-2^ d^-1^[28]. Craggs et al. [29] provide a good summary of production in HRAP. There is a wide variability of production rates achieved based on wastewater source, type, location and culture conditions. Algae growth in HRAPs has also been shown to achieve greater than 75% nutrient removal [30]. Production was shown to improve with CO_2_ addition from 10.6 to 15.2 g dry wt m^-2^ d^-1^. Li et al. [20] and Zhout et al. [21] scaled up their wastewater-grown algal with 25-L BIOCOIL reactors and obtained net biomass productivity of 13 and 12.8 g dry wt m^-2^ d^-1^ respectively.

**Figure 7:**
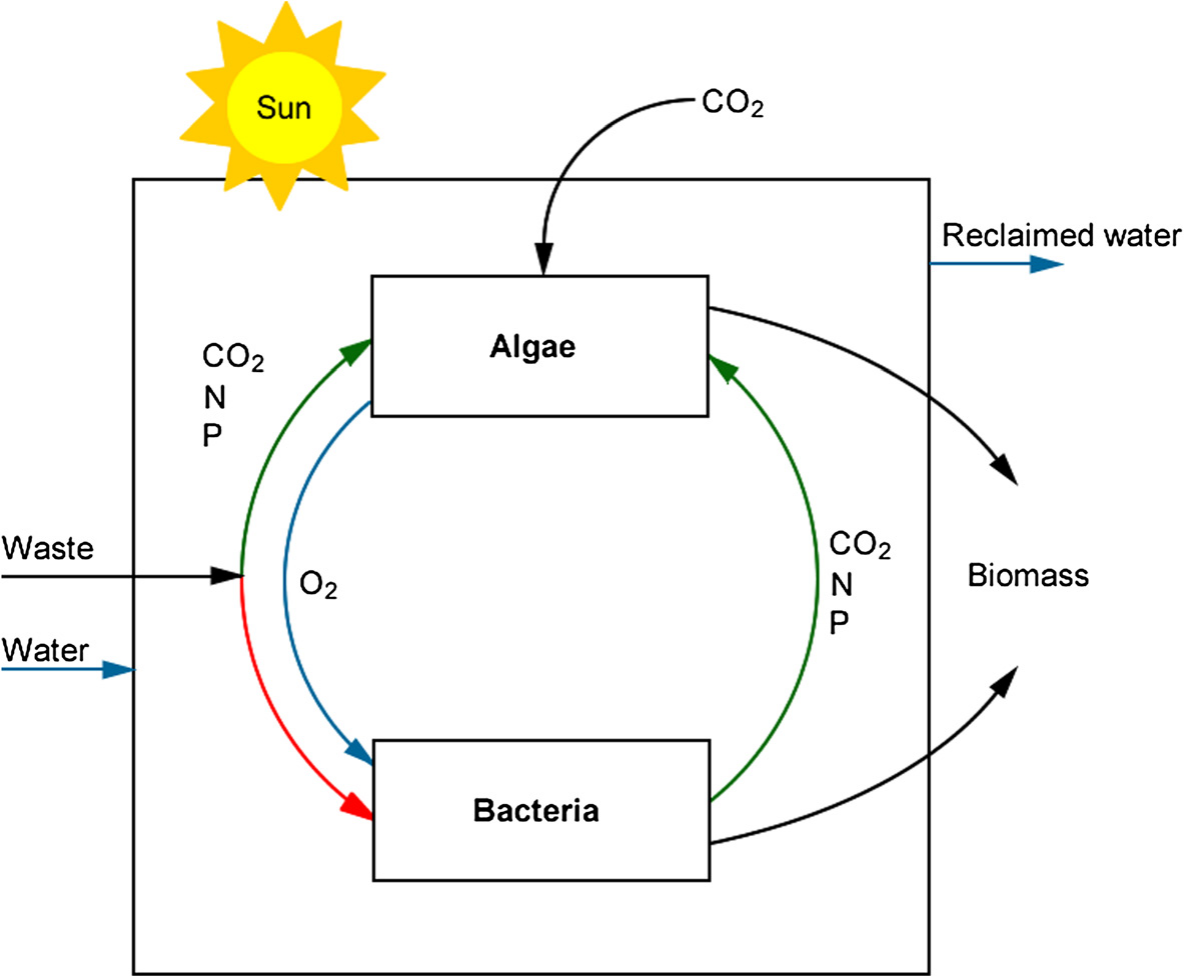
**Basic operation principles for the algal production integration with wastewater treatment [**[10]**].**

**Figure 8:**
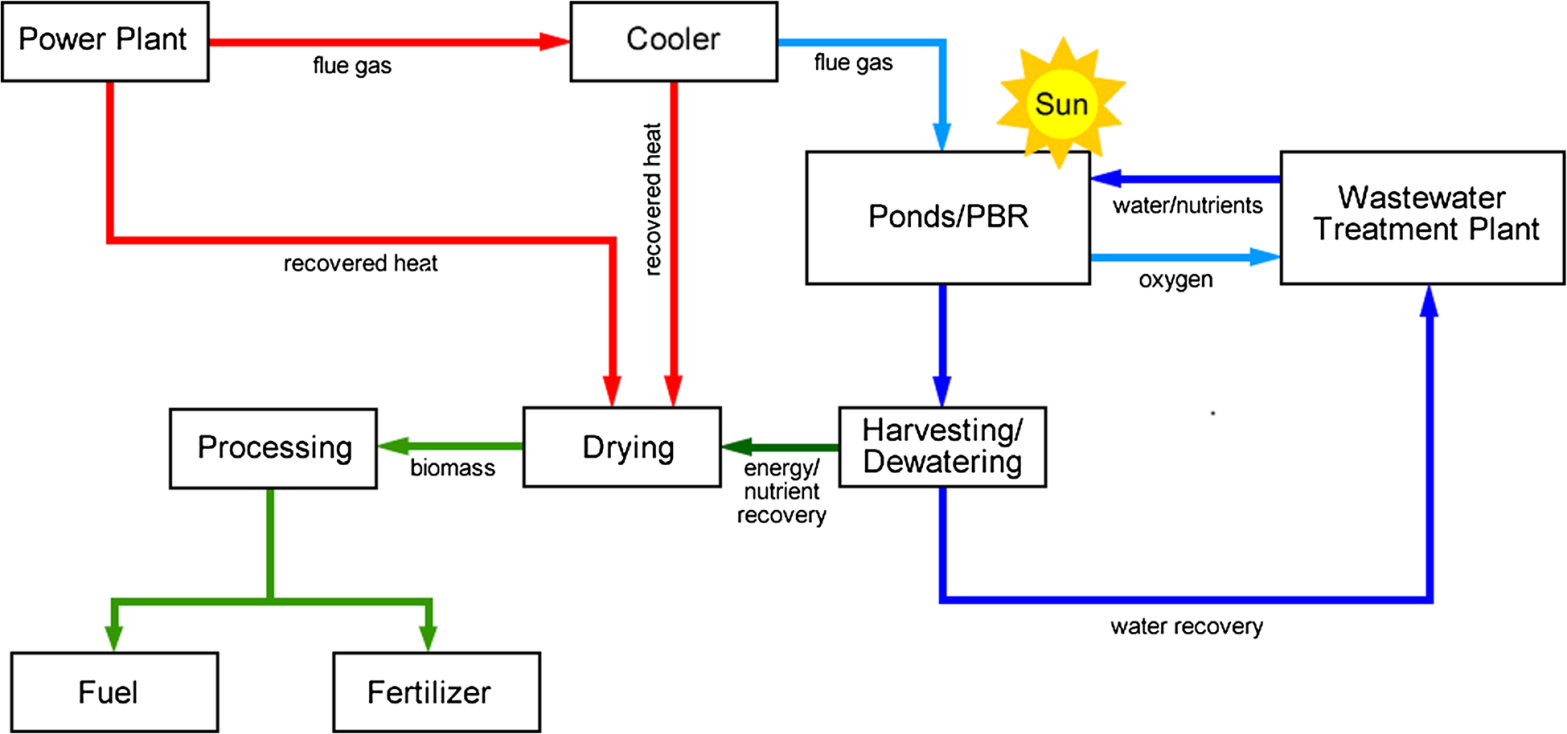
Algae production integration with power generation and wastewater treatment.

The basic principles and a schematic behind the operation for algal integration with wastewater facilities and power plants are shown in Figure 5 and Figures 7 and 8. While the challenges associated with algal harvesting, species control, and fuel conversion must be solved for large-scale production, the harvestable yields of algal biomass (g dry wt m^-2^ d^-1^) helps to determine the potential of algal systems for energy and fuel production. These yields depend largely on nutrient availability and lighting conditions. In this section, the nutrient removal efficiency and observed areal productivity for the bench-scale photobioreactors are used to determine the size of the algae production facility.

Microalgae biomass results mainly from photosynthesis, which utilizes inorganic compounds (including CO_2_). In simple terms, algal biosynthesis can be described by the following chemical equations where ammonium and nitrate are the nitrogen sources respectively [31,32];

(3) $$\begin{array}{l} 16NH_{4}^{+}+92CO_{2}+92H_{2}O+14\mathrm{HC}O_{3}^{-}\\ \;+\mathrm{HP}O_{4}^{2-}\overset{\mathrm{hv}}{\to }C_{106}H_{263}O_{110}N_{16}P+106O_{2} \end{array}$$

(4) $$\begin{array}{l} 16NO_{3}^{-}+124CO_{2}+140H_{2}O\\ \;+\mathrm{HP}O_{4}^{2-}\overset{\mathrm{hv}}{\to }C_{106}H_{263}O_{110}N_{16}P+138O_{2}\\ \;+18\mathrm{HC}O_{3}^{-} \end{array}$$

In the above equations, the chemical formula C_106_H_263_O_110_N_16_ represents algal biomass [32]. According to the stoichiometry, 1 g of ammonia-nitrogen (NH_3_-N) or nitrate-nitrogen (*NO*_3_^−^ − *N*) produces about 15.8 g of biomass and consumes 18.1 and 24.34 g of CO_2_ in the process, respectively. In addition to nutrient availability, algal biomass production also depends on light energy (*hv*). In the absence of nutrient limitation, photosynthesis increases with increasing irradiance until the maximum algal growth rate is attained as described my Michaelis-Menten kinetics [24-26]. A condition known as photoinhibition can occur when the irradiance is increased beyond the saturation point resulting in damage to algal light receptors and a decrease in the photosynthetic rate and productivity [24,25].

The total amount of algal biomass produced may be estimated by considering the total flows of nitrogen. Nitrogen is assumed to be the limiting nutrient since phosphorous is generally considered to be an abundant nutrient in Tampa due to the numerous phosphate deposits. The annual production estimates for algal production based on the concentrations of nitrogen in wastewater from the HFC AWTP and the Lakeland WTS are shown in Table 2. These calculations include the average flow rate of water passing through each plant. The required area to facilitate production is estimated based on the observed productivity for algae grown on centrate and the Lakeland WTS water. The growth rate and lipid production for algae grown on wastewater with moderate nitrogen levels (~30 mg/L) were adopted from Woertz et al. [19] as 3 g dry wt m^-2^ d^-1^ and 30% lipids by dry weight respectively.

**Table 2:** Potential biomass production estimates for algae grown on wastewater nutrients in the Tampa Bay area, FL

**Description**	**Source**	**Flow rate (MGD)**	**Nitrogen (mg L**^**-1**^**)**	**Algae biomass (tons yr**^**-1**^**)**	**CO**_**2**_**consumed (tons yr**^**-1**^**)**	**Indoor area (ha)**	**Outdoor area (ha)**
Wastewater	HFC AWTP	3.0	30	1,965	3,026	179	179
Centrate	HFC AWTP	0.5	427	4,660	7,179	182	80
Wastewater	WTS	5.0	10	1,091	1,681	598	598
**Total**				**7,716**	**11,889**	**959**	**857**

Algal production is restricted by available land close to the HFC AWTP. Approximately 200 hectares of suitable land area is available onsite and is located within close vicinity to where centrate is generated. Therefore, the flow rate has been chosen to reflect the land restriction for the indoor production. It is assumed that algae grown on moderate and low strength nutrient are nutrient limited and hence, their productivities are not affected by increasing light beyond a certain value. However, for algae grown on high strength centrate, the outdoor production area can be reduced since the algae are not nutrient limited.

### Energy production and revenue potential

#### Liquid biofuels

For biofuel production, algae need to have a lipid content exceeding 20% [10], some researchers even suggest 40% [33]. This means that high strength wastewater would not be suitable for cultivating algae for lipid production. Realistically, the best algae for lipid production are those from the Lakeland WTS or algae grown on low strength wastewater. Usable lipids were assumed to be 20% and 50% of the algae dry wt. for moderate strength wastewater and low strength pond water, respectively. An algal oil-to-biofuel conversion efficiency of 80% was used, which is similar to that obtained for vegetable oil [34]. The biofuel potential for the various algae are shown in Table 3. The total potential volume of biofuel obtained is approximately 269,545, which can, on average, fuel 450 cars per year (assuming 15,000 miles yr^-1^ with an average of 25 miles per gallon).

**Table 3:** Annual biofuel production estimates derived from algae growth in wastewater nutrients in the Tampa Bay area, FL

**Description**	**Source**	**Algae biomass (tons yr**^**-1**^**)**	**Biofuel (gal yr**^**-1**^**)**	**Total revenue (US$ yr**^**-1**^**)**^**1**^
Wastewater	HFC AWTP	1,965	112,833	451,332
Wastewater	WTS	1,091	156,712	626,848
**Total**		**3,056**	**269,545**	**1,078,180**

^1^Biodiesel cost $4.05 per gal taken from the Center for Agricultural and Rural Development (CARD), http://www.card.iastate.edu/research/bio/tools/hist_bio_gm.aspx, Iowa State University, Ames, Iowa.

#### Biogas generation

Algae biomass may be anaerobically digested to produce methane, especially biomass which may be considered unsuitable for liquid biofuel production due to low lipid content. The stoichiometric relationships for this process are illustrated in Equation (5), which were developed from half reactions assuming that ammonia is the nitrogen source [35]. The fraction of electrons towards energy production (f_e_) was estimated to be 0.89 based on the work by Yuan et al. [36].

(5) $$\begin{array}{l} C_{106}H_{263}O_{110}N_{16}+6.672H2O\\ \to 13.668NH_{4}^{+}+33.502CO_{2}+47.170CH4\\ +2.332C_{5}O_{2}H_{7}N+13.668HCO_{3}^{-}+HPO_{4}^{2-}+2H^{+} \end{array}$$

According to equation 5, 1 mole of algae biomass produces 47.17 moles of methane. However, previous research has shown that algal biomass is not particularly easy to digest having a biogas yield of 29.5% [36,37]. Therefore, 1 g of algae dry wt. is estimated to generate 62.7 mg methane. The estimated production of biogas and the derived energy are shown in Table 4 assuming that the energy content of methane is 55 MJ kg^-1^ for the HFC AWTP.

**Table 4:** Biogas production estimates for anaerobic digestion of algae biomass grown on wastewater nutrients

**Description**	**Source**	**Algae biomass (tons yr**^**-1**^**)**	**Biogas production (kg yr**^**-1**^**)**	**Total energy (MJ yr**^**-1**^**)**	**Households powered**^**1**^
Wastewater	HFC AWTP	1,965	123,215	6,776,838	144
Centrate	HFC AWTP	4,660	292,294	16,076,165	342
**Total**		**6,625**	**415,509**	**22,853,003**	**486**

^**1**^ Assuming average energy consumption of 653 kWh per month and thermal efficiency of natural gas turbine of 60% with waste heat recovery.

The above calculations assumed that the total production of algae goes toward digestion. It is also possible to extract lipids and attempt to derive biogas from spent biomass. The combination of algae production on the wastewater nutrient sources shows the potential for energy generation that can power close to 500 homes.

## Conclusions

This work shows that there are important benefits to be derived from integrating algal production systems with nutrient-rich waste streams. The feedstock potential of the HFC AWTP and the Lakeland WTS is estimated to be approximately 71 tons ha^-1^ yr^-1^ of algal biomass, 270,000 gal hr^-1^ of liquid biofuel, and 415,000 kg yr^-1^ of methane. Renewable energy derived from algae will play a significant role in providing energy security while important services such as water treatment can be synergistically achieved by these systems. Even though the analysis has been preliminary, it shows that there is good potential for algal feedstock production in the Tampa Bay area. However, there are many important factors to be considered to assess whether algal production systems would be competitive. These include analysis of energy and cost associated with harvesting and extraction for example. It is hoped that with further research many of these challenges can be overcome.

## Competing interests

The authors declare that they have no competing interests.

## Authors’ contributions

OKD, TH, IU and BG designed, constructed and operated the photobioreactors and carried out all algae and nutrient analysis related to the centrate studies OKD and TH conducted the studies based on the Lakeland algae samples, carried out lipid analysis and drafted the manuscript. SE, JW and QZ were co-PIs on this project and advised the research assistants on this project. All authors read and approved the final manuscript.
